# Cryopreservation of Endangered Ornamental Plants and Fruit Crops from Tropical and Subtropical Regions

**DOI:** 10.3390/biology11060847

**Published:** 2022-05-31

**Authors:** Behzad Kaviani, Dariusz Kulus

**Affiliations:** 1 Department of Horticultural Science, Rasht Branch, Islamic Azad University, Rasht 4147654919, Iran; b.kaviani@yahoo.com; 2 Laboratory of Ornamental Plants and Vegetable Crops, Faculty of Agriculture and Biotechnology, Bydgoszcz University of Science and Technology, Bernardyńska 6, 85-029 Bydgoszcz, Poland

**Keywords:** cryo-plate, cryo-mesh, genetic resources, genetic stability, in vitro conservation, omics technologies, regeneration, stress

## Abstract

**Simple Summary:**

The protection of biodiversity, i.e., the biological variety and variability of life on Earth, is of great importance for the present and future generations. Maintaining variation at the genetic and ecosystem levels is indispensable in breeding programs and creation of new cultivars. Currently, numerous plant species, wild varieties, and local forms of ornamental and fruit plants are endangered with extinction. Cryopreservation, i.e., the storage of biological samples in tanks filled with liquid nitrogen is considered as the most effective long-term preservation method of plant genetic resources. Nonetheless, the establishment of efficient cryogenic procedures is a difficult task, requiring consideration of several factors. The impact of cryopreservation on the stability and homogeneity of the stored samples is of particular interest. The aim of this article is to evaluate some traditional and modern cryopreservation methods and their utility for the storage and exchange of genetic sources of tropical and subtropical horticultural crops.

**Abstract:**

Horticultural crops comprise various economic species extending from fruits, nuts, vegetables, spices and condiments, ornamentals, aromatic, and medicinal plants. Ornamental and fruit plants are produced mainly for their nutritional and aesthetic values, respectively. Unfortunately, many tropical and subtropical species are in danger of extinction because of climate change and (a)biotic stresses. It is imperative to preserve the germplasms of these species for the present and future genetic improvement programs. Cryopreservation, i.e., maintenance of tissues at the ultralow temperature of liquid nitrogen, is a promising long-term preservation technique, alternative to seed or in vitro banks, which can be applied for both vegetatively and generatively (through seeds) propagated crops, including those with recalcitrant seeds. It is a technology of choice not only for the preservation of plant biodiversity but also for virus elimination in the proficient administration of large-scale micropropagation. The main advantages of cryopreservation are the lowering of in vitro culture expenditures, needed space, contamination risk, and operator errors. However, tropical species are temperature delicate and one of the foremost challenging issues is preconditioning treatments that stimulate physiological reactions to sufficiently enhance tolerance to dehydration and cryogenic procedures. In recent years, several cryopreservation methods based on encapsulation-vitrification, droplet-vitrification, the use of aluminum cryo-plates, and cryo-mesh have been established. Combined cryo-techniques, gene/DNA conservation, as well as studies on perceiving bio-molecular events and exploring the multistage process from the beginning to end of cryopreservation are receiving more emphasis. The development of cryobiomics delivers a conceptual framework to assess the significance of cell signaling mechanisms on cellular functions, the influence of cryoinjury factors on sample viability, and the implications for genetic stability following cryo-storage. The aim of this mini-review article is to provide a succinct synthesis of the developed cryogenic procedures and their use for the storage and exchange of genetic resources of tropical and subtropical horticultural crops, particularly fruit crops and ornamental plants under the threat of extinction.

## 1. Introduction

It is estimated that approximately 13% of higher plants are on the brink of extinction. Many of those endangered species are fruit and ornamental plants. Several environmental and endogenous factors alter the level of plant biodiversity in natural populations, which is the basis of evolution and adaptation [1]. The rapid development of proper approaches for the long-term preservation of genetic resources seems to be necessary. Germplasm is a live information source for all the genes present in the respective plant, which can be conserved for long time spans and regenerated whenever it is required (particularly for crop breeding and improvement) [2,3]. Conventional in situ and ex situ conservation of germplasms through seed storage, clonal means, gene banks, national parks, sanctuaries, natural habitats, botanical gardens, and cold storage have some limitations regarding efficacy, genetic erosion, security, costs, and long-term maintenance [4]. Conservation of plant biodiversity through innovative biotechnology techniques includes cryopreservation.

Cryopreservation refers to the storage of tissues in liquid nitrogen (LN, −196 °C) or, less frequently, LN vapor (approximately −165 to −190 °C) [5,6]. In LN, the metabolic and biochemical processes (including respiration and enzymatic activity that would lead to cell aging and cell death), as well as cell division are effectively arrested, which makes this long-term storage possible [5,7,8,9]. Cryopreservation is the only feasible technique for the long-term preservation of genetic material from different categories of plants, i.e., non-orthodox (dehydration-sensitive) seed species, vegetatively propagated plants, rare and endangered species, as well as valuable plant cell lines [8,10,11,12,13]. 

Fast and easy access to high-quality gene banks is the key for breeders and producers. Currently, cryopreservation procedures are available for more than 200 plant species and over 10,000 accessions started from in vitro cultures are safely stored in LN [5]. Over 80% of these belong to five major crops: i.e., potato (38%), cassava (22%), banana and plantains (11%), mulberry (12%), and garlic (5%) [14]. Other vital cryostored plant collections, representing thousands of accessions, are those of dormant apple buds [5]. At the same time, about half of the world’s top 10 endangered plant species are ornamentals. Among the endangered ornamental plants, one can mention the following genera: Fritillaria, Buxus, Lilium, Taxus, Agave, cycads, Zamia, bromeliads, pines, orchids, and many others. Some of these species have medicinal properties in addition to aesthetic value. To date, cryopreservation has been successfully used for several ornamental and fruit plants [5,12,15,16]. The first report on cryostorage of those species was described by Fukai [17] on carnation hybrid and by Sakai [18] on mulberry, respectively.

Various types of explants can be cryopreserved, including mature seeds, apical or axillary shoot tips, embryogenic cultures, pollen, zygotic and somatic embryos, embryonic axes, shoot primordia, roots, protocorms, protocorm-like bodies (PLBs), cell suspensions, callus, protoplasts, spores, bulblets, tiny leaf square-bearing adventitious buds (SLS-BABs), stem disc-bearing adventitious buds (SD-BABs), microtubers induced from nodal segments, rhizome buds, dormant buds, plumules, and in vitro derived explants [1,15]. Among them, shoot tips are used most often with tropical and subtropical fruit and ornamental species. On the other hand, the LN-storage of embryogenic cultures is a vital tool that supports the advancement of breeding programs based on somatic hybridization [19]. LN-recovered embryogenic tissues have been also applied for genetic transformation in a great number of economically significant (sub)tropical crops, e.g., banana, cassava, and citrus [20]. 

Cryopreservation, i.e., the whole in vitro culture–cryoprotection–regeneration process, results in the exposure of cells to chemical, physical, and physiological stress-causing cryoinjury, though its impact on the genome is often unidentified [21]. Therefore, it is essential to verify that the genotype and/or phenotype of cryopreserved germplasm are true-to-type [22]. Cryobiomics is a novel study of cryoinjury, genetic stability, dynamics, and behavior of cryopreserved cells, tissues, or organisms [21]. Advances in cryobiomics’ technologies facilitate the development of robust cryopreservation procedures. Those studies connect causal factors related to cryoinjury and loss of explant viability to the risks of genetic instability [22]. 

The objective of this article is to evaluate some traditional and modern cryopreservation methods and their utility for the storage and exchange of genetic sources of tropical and subtropical horticultural crops, particularly fruit and ornamental plants under the threat of extinction. The article also highlights the general principles adapted for cryopreservation of plant tissues by using omics technology.

## 2. Tropical and Subtropical Ornamental and Fruit Plants

Tropical and subtropical plants are grown in the equatorial zones of the world. Horticulture in those parts of the world includes perennial woody plants (arboriculture), fruits (pomology), vegetables (olericulture), and ornamentals (floriculture). The origin of many of these crops is in the temperate regions and their adoption to tropical and subtropical climate conditions is a goal of breeding. Several important crops, however, are indigenous to the tropics and subtropics. Since the tropical zone represents 36% of the Earth’s surface (and 20% of its land surface), the potential of tropical horticulture is noticeable. 

The ornamental plants sector is developing at the global level, in both production and trade [23]. In 2019, the value of the floricultural market on the largest global flower exchange, Royal FloraHolland, reached EUR 4.8 billion. In 2021, despite the problems related to the global pandemics of SARS-CoV-2, the annual value of the flower trade increased to EUR 5.6 billion and it is predicted to continue expanding [24]. Many of the most popular ornamentals are endangered tropical plants, such as orchids or members of the Araceae, Heliconiaceae, or Bromeliaceae families [13].

**Figure 1:**
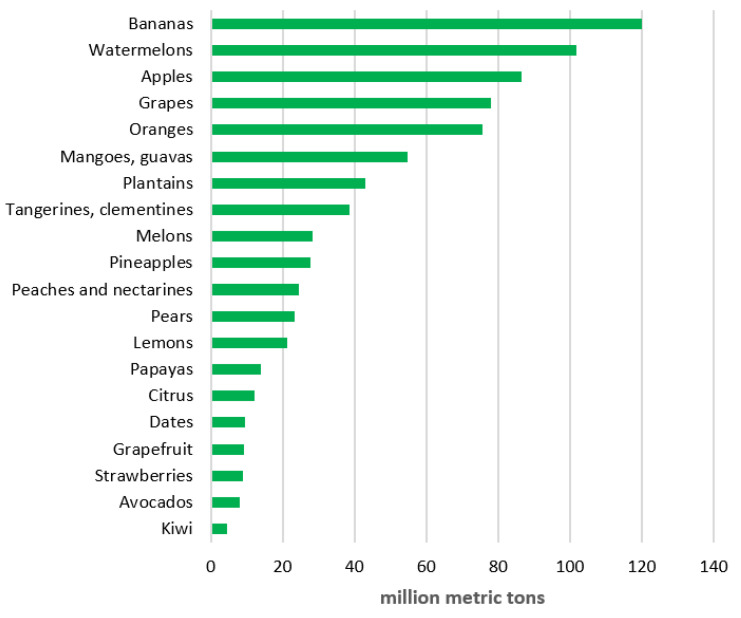
Global fruit production in 2020 [28].

As for the tropical and subtropical fruits, most of them have nutritional, medicinal, and industrial properties [25,26]. Their production has been increasing significantly faster than that of temperate fruits in recent years (Figure 1). Unfortunately, many of the (old) cultivars and landraces of the most important tropical and subtropical fruit plants can perish [13,27]. The reason for their dithering numbers comes down to climate change, land development, urbanization, and deforestation. Therefore, it is of utmost significance to establish effective preservation procedures for those genetic resources.

## 3. Importance and General Principles of Cryopreservation

Among some of the most significant advantages of cryopreservation, one can mention: preserving the genetic diversity by storing wild and endangered species (especially for breeding), eliminating viruses from infested plants by shoot apex cryotherapy, minimum storage requirements, maintenance of phenotype and genotype stability, safe conditions from diseases or damage triggered by the environment, negating the need for continual and serial sub-culturing, and facilitating the international germplasm exchange [13,19].

However, the development of effective cryogenic procedures is not an easy task. Problems caused by exposing the biological material to low and ultra-low temperatures include effects on all kinds of processes, i.e., slow down or stop of the biochemical reactions (and thus the metabolism), instability and loss of semi-permeability of membranes, breaking of tissues, and disconnection of cells, non-reversible plasmolysis and, finally, cell death [29,30]. Avoidance of chemical and physical injuries during cryopreservation is the main objective of cryoprotection [31]. Extracellular ice formation can be harmful to the integrity of cellular structures, but intracellular ice is lethal. Therefore, in all cryopreservation procedures, the removal of water plays a key role in avoiding freezing damage and in securing the *post*-thaw viability of LN-derived samples [32]. This can be obtained with the use of the so-called cryoprotectants. Cryoprotectors are chemical compounds that interact and modulate water distribution inside/outside cells and dehydrate them [33]. These substances enhance the stability of the plasma membrane, lower the freezing point and increase the viscosity of the cytosol and, at the same time, protect the cells from injury throughout cooling. There are two main types of cryoprotectants: (1) penetrating cryoprotectants to the cell, mainly with colligative properties (such as DMSO, glycerol, EG, and propylene glycol-PG); and (2) non-penetrating cryoprotectants with osmotic activity (such as sucrose, dextrans, and proline) [34]. ‘Colligative acting’ is a more accurate term to describe both penetrating and non-cell-penetrating cryoprotectants that can alter solute concentration during cryopreservation [35]. In some types of tropical and subtropical plant germplasms, a mixture of both cell-penetrating and non-penetrating cryoprotectants can be employed [31,36,37], although the effect of cryoprotectors, including their permeability and toxicity, is species-specific.

The efficiency of any cryopreservation method is highly determined by balancing the plant’s intrinsic tolerance to stress with the capability to endure cryogenic treatments that must be optimized [32]. Generally, six critical factors need to be considered when developing a cryoprotocol: 1. pretreatment, manipulating the in vitro culture conditions to increase the explant’s tolerance to low temperatures; 2. treatment with osmoregulating and cryoprotecting compounds to enable the cells to withstand freezing, the precautions are mainly associated with the toxicity of cryoprotectants; 3. cooling or freezing (slowly or rapidly); 4. storage in LN; 5. rewarming of the stored material; and 6. recovery, which evaluates the viability of the biological material. A cryopreserved collection may be used for the establishment of a safety backup for crops propagated clonally. An additional benefit of such backup, compared to the traditional seed bank, is that it can serve hundreds or even thousands of years and does not require regeneration after a few years of storage [38]. However, despite cryogenic storage having evident strategic merit, the resulting injuries from the cooling and rewarming cycles (affecting the membranes structure, cellular functions, and recovery potential) can cause unacceptable declines in the cell’s viability and regrowth of complete plants, which remains a major limiting factor [32]. Chimerism, i.e., composition of cells with more than one distinct genotype, is an additional difficulty in the long-term storage of ornamental plants [30].

## 4. Cryopreservation of Endangered Ornamentals and Fruit Crops

Cryopreservation can become the most efficient strategy for the safe long-term maintenance of rare and endangered ornamental plants [15,16]. It is also useful with tropical and subtropical fruit species, especially those that produce desiccation-sensitive, recalcitrant (non-storable) or intermediate seeds (e.g., cocoa, citrus, cacao, coffee, coconut, avocado), root and tuber crops, sterile crops that do not produce seeds (banana), or species which are predominantly propagated clonally (e.g., cassava), as well rare and overexploited species [8,39,40,41]. It is estimated that approximately 100,000 unique accessions of clonally propagated fruit tree species and recalcitrant seed crops require long-term preservation through cryostorage, while currently only around 10,000 accessions are stored in LN [14]. Therefore, more work in this area is needed.

Tropical plants do not have special protective mechanisms that allow them to survive at sub-zero temperatures [15]. Therefore, cryopreservation of these species is a critical process and some criteria (such as the content of intracellular water, applied cryopreservation method, and the difficulties with explant handling) should be considered with particular scrupulosity. Step-by-step optimization of cryopreservation techniques is fundamental to achieving satisfying results, i.e., at least 30–40% recovery of the LN-stored specimens. Two types of cryopreservation protocols can be distinguished that vary in their physical mechanisms: (i) conventional (classical) or two-step procedures, in which cooling takes place slowly in the presence of ice (based on freeze-drying); and (ii) modern or one-step procedures based on vitrification, during which rapid cooling is performed without ice formation [42]. Cryopreservation protocols of some important tropical and subtropical ornamental and fruit species by various techniques are presented in Table 1 and Table 2.

### 4.1. Conventional Methods (Two-Step Cooling)

**Figure 2:**
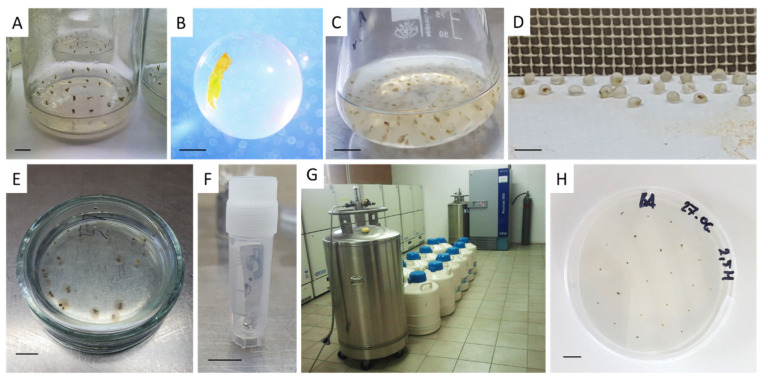
Cryopreservation steps of the encapsulation-dehydration, encapsulation-vitrification, and droplet-vitrification techniques: (**A**) preculture of nodal explants on the medium with an increased sucrose and ABA concentration; (**B**) shoot tip encapsulated in calcium alginate; (**C**) osmotic dehydration of encapsulated explants in a concentrated sucrose solution; (**D**) air drying of explants; (**E**) dehydration in PVS; (**F**) cryotube with aluminum foil strip and attached shoot tip; (**G**) cryobank in the Botanical Garden of the Polish Academy of Sciences in Warsaw, Poland; (**H**) recovery of explants on a cytokinin-supplemented medium. Bar = 1 cm (except for Figure 2(**B**)—1 mm).

**Figure 3:**
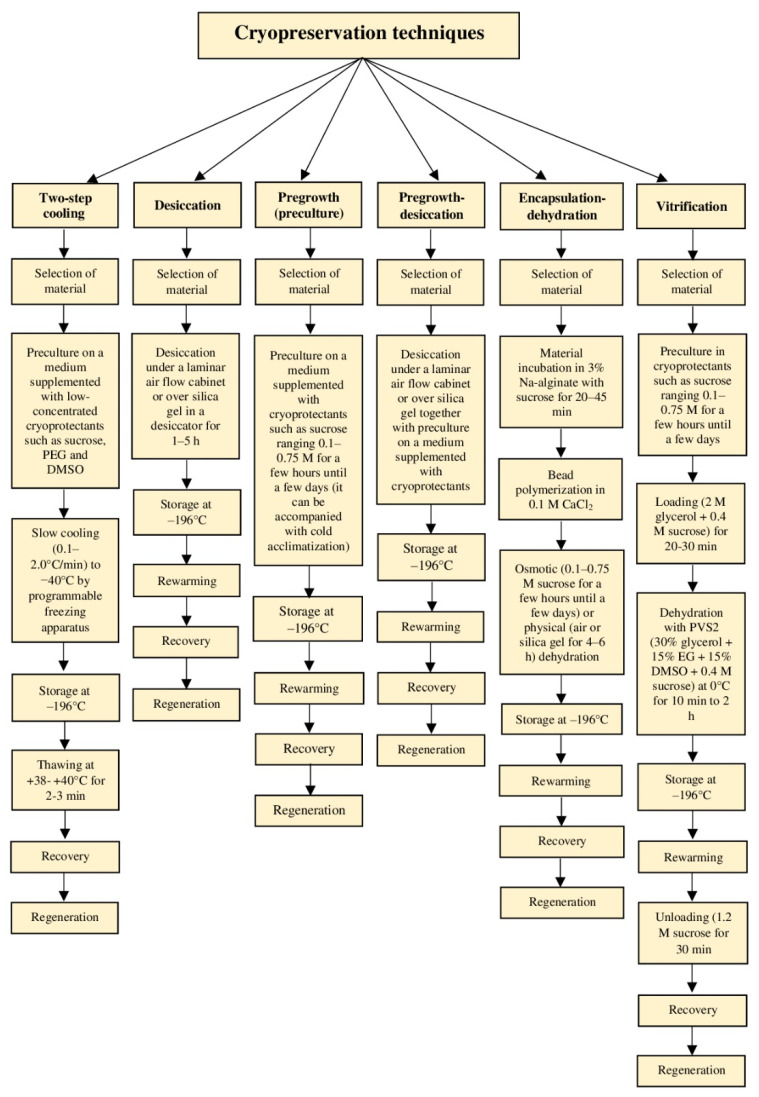

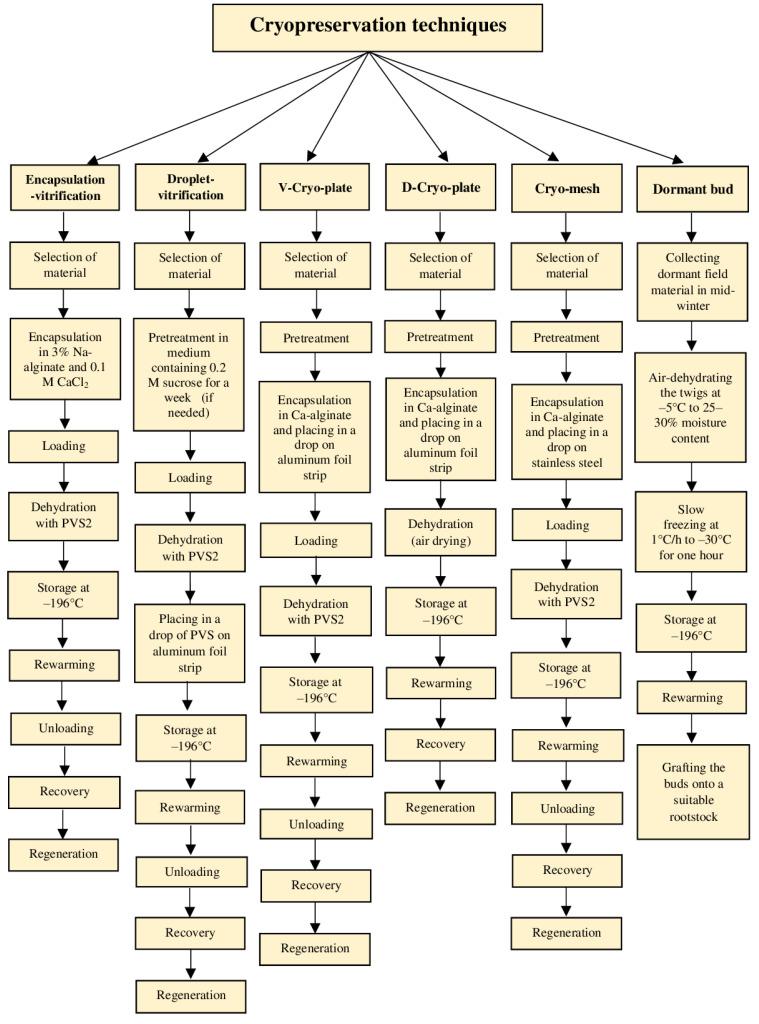
Schematic representation of different cryopreservation protocols.

Various methods are used to lower the temperature for cryogenic storage depending on the cooling rate (Figure 2 and Figure 3); ultra-rapid, rapid, and slow cooling [19]. A programmable freezing apparatus is used most often in the slow cooling technique (two-step or controlled-rate freezing) to obtain precise and reproducible thermal conditions. During the slow temperature decrease, ice is primarily formed in the extracellular solution that promotes the efflux of water from the cytoplasm and vacuoles to the outside compartments of the cell where it finally freezes [19]. This freeze-dehydration aims at promoting the formation of an amorphous semi-solid-state in the cells if the amount of remaining intracellular water at the moment of plunging the specimens in LN is so low that it vitrifies [19,43]. Classical methods involve the pretreatment of biological material with solutions composed of a single or a mixture of cryoprotective substances (usually DMSO), proceeded by the slow cooling (0.1–2.0 °C·min^−1^) down to a pre-freezing temperature (usually −40 °C) prior to rapid immersion of samples packed in straw or cryotube in LN [15,19]. The rate of the temperature decrease in LN is in the range of 1000 °C·min^−1^. Studies on over 700 cell lines from around 600 plant species revealed that this approach is most successful with undifferentiated materials, i.e., callus and cell suspension [15,44]. Among more differentiated explants, only the shoot tips of species tolerant to low temperatures can be cryoconserved with this technique [45]. Effective cryopreservation of shoot tips from tropical plant species (such as cassava) is an exceptional example [46]. Moreover, two-step freezing is expensive and, therefore, is becoming less and less used [15].

### 4.2. Modern Methods (One-Step Cooling or Vitrification-Based Methods)

The only way to prevent ice crystallization in the cell at ultra-low temperatures of LN without an extreme reduction in water content is by vitrification, i.e., *quasi-*solidification of a solution into a “glassy” state [5]. Vitrification can be obtained by increasing the viscosity of a cell to the critical point at which ice formation is hindered; both inside and outside the cell, when the available residual water becomes vitrified on exposure to liquid nitrogen [31]. All modern cryogenic approaches rely on this phase transition.

Rapid and ultra-rapid cooling rates are typical for vitrification-based cryoprotocols that involve some level of dehydration by exposure of tissues to highly concentrated solutions of cryoprotectants and/or too strong physical drying (desiccation) [19]. Consequently, most or all freezable water is removed, and the internal solutes vitrify when the explant is plunged into LN [46]. A plant vitrification solution (PVS), which is a mixture of two, three, or four cryoprotectors, provides a transition of the remaining intracellular water to the amorphous “biological glass” state [6]. Different modern methods (vitrification, droplet-vitrification, encapsulation-dehydration, encapsulation-vitrification, and cryo-plates) have been adapted for the cryopreservation of complex organs such as shoot tips/apices and somatic embryos [19] (Figure 3). Most of these methods have been derived from the two most basic cryogenic strategies, vitrification, and encapsulation-dehydration [13].

A typical vitrification-based protocol consists of explant preculture, encapsulation, and/or treatment with a loading solution (LS—a mixture of diluted non-toxic cryoprotective agents), proceeded by either dehydration with PVS or desiccation, and then rapid immersion in liquid nitrogen. After rewarming and washing off the cryoprotectors, explants are transferred to a recovery medium [15]. The use of modern techniques is increasing steadily in genebanks worldwide, particularly for the improvement in the cryopreservation of tropical and subtropical fruit and ornamental plant germplasms [13]. Modern techniques are superior to slow cooling as they allow for the storage of more differentiated plant materials of both cold- and desiccation-susceptible/tolerant species, guarantee higher cooling rates, and are cheaper than the traditional techniques [15].

#### 4.2.1. Desiccation

Desiccation is the easiest cryopreservation technique and consists of explant dehydration and, then, their direct plunge in LN [10]. Meristematic cells with only a few small vacuoles and relatively low water content show high tolerance to ultra-freezing, while the cells containing large vacuoles do not survive and must be dehydrated. Desiccation or drying of the plant samples is performed under a stream of sterile air (in an air-flow cabinet) or, more precisely, over a silica gel (in a desiccator) for 1–5 h (possibly preceded by osmotic dehydration in a sucrose solution), prior to rapid immersion in liquid nitrogen (Figure 3). Sometimes, desiccation is the sole requirement for establishing a successful cryoprotocol [15]. However, the simplicity of this technique is accompanied by its limited use. The method can be applied for pollen, embryos, orthodox seeds, and the shoot tips of desiccation-resistant plants [15], e.g., some endangered, rare, ancient, and wild *Citrus* species [47].

#### 4.2.2. Pregrowth or Preculture

The pregrowth technique (Figure 2a and Figure 3) consists of in vitro culturing the explants in media containing sugars (sucrose, glucose, and fructose) or sugar alcohols (mannitol and sorbitol) proceeded by exposure to cryoprotectants and rapid immersion in LN [10]. Preculturing the explants is often the first and fundamental step in several cryoprotocols developed for ornamental plants and in cold-sensitive tropical and subtropical fruit species. It is an important factor in the cryostorage of gentians, some lilies, and orchids [15,48]. Preculture stimulates the accumulation of endogenous sugars, polypeptides, abscisic acid (ABA), proline, and bounded water, which enhance the plant’s tolerance to stress [49]. Medium composition, culture duration, and exposure temperature to osmotically active substances are the critical factors for successful cryopreservation [50]. Sucrose (mainly 0.3–0.5 M) applied for a few hours until a few days is the most commonly applied to induce cryo-tolerance and osmotic dehydration. However, extremely strong dehydration can result in the death of a cell as a result of progressing plasmolysis and osmotic shock, as reported with *Vanilla planifolia* Andrews [51,52]. Using a two-step preculture with increasing sugar content may minimize this adverse effect [15]. Besides sugar addition, low temperature (0–5 °C) and light quantity in the growth room, as well as application of some exogenous compounds such as ABA and proline can be effectively used. These factors stimulate the hardening of plants and alter the dynamics of water crystallization by lowering its freezing point and preventing ice nucleation [15].

#### 4.2.3. Pregrowth-Desiccation

Pregrowth-desiccation (Figure 3) is a combination of the preculture and desiccation methods. The use of an appropriate concentration of cryoprotectants in the culture medium protects the plant cell from hydrodynamic damage and cryoinjury. This technique is primarily used for the cryostorage of meristems, small-sized seeds, zygotic embryos, polyembryonic cultures, or embryonic axes [13].

#### 4.2.4. Encapsulation-Dehydration

The encapsulation-dehydration technique (Figure 2b–d and Figure 3) is based on the technology developed in the 1970s for the production of manufactured (artificial/synthetic) seeds. Plant material is encapsulated in alginate beads (mainly sodium alginate), dehydrated in a liquid medium enriched with sucrose, partially desiccated to a water content of around 20–30%, and then plunged rapidly in LN [13]. A standard encapsulation-dehydration procedure involves the incubation of explants in 2–4% alginate (10 min), proceeded by bead polymerization in 0.1 M CaCl_2_ (20–45 min), and dehydration (osmotic and physical) [15]. Preferably, 0.1–0.75 M sucrose can be used for a few hours until a few days for rapid or gradual osmotic dehydration. The optimal physical dehydration (drying) duration is 4–6 h. Concentration and incubation time during the following steps may be manipulated. The presence of alginate bead reduces the pace of the dehydration process and allows the application of subsequent dehydration processes to lower the cells’ moisture content before LN-storage, which would be highly damaging or even lethal to non-encapsulated explants [13,53]. Moreover, the alginate matrix provides enhanced physical protection of the samples from mechanical and oxidative stress during storage and ease of handling during pre- and post-LN-storage steps [54]. The presence of sucrose in the capsule, besides dehydration, may stimulate faster recovery of the explants after rewarming [55]. The addition of some secondary metabolites into the capsule, e.g., salicylic acid (SA), may also greatly improve the protocol success [7,56,57]. Adding glycerol into the bead matrix, on the other hand, was effective for *Dendrobium nobile* PLBs [58]. Several reports highlighted that encapsulation-dehydration may provide better protection than PVS or sucrose pretreatment alone [59,60,61,62]. This technique is the most frequently used with ornamental plants [12,15,16,63]. It is widely used, for example, with orchids (seeds and PLBs) [64,65]. Encapsulation-dehydration has been also employed to *Arabidopsis* and shoot tips of some fruit tree species including apple, pear, and *Prunus* [66,67]. Moreover, this technique is highly effective with the storage of cell suspensions and calli [51]. Despite some advantages, the encapsulation-dehydration method is time-consuming, and coating the material in an alginate matrix increases the risk of hyperhydricity of the tissues and callus regeneration after LN-storage [15,60]. Therefore, other cryopreservation techniques are also used.

#### 4.2.5. Vitrification

The vitrification technique (Figure 3) has been employed for more than 100 plant species and is now the most widely applied plant cryopreservation protocol, especially with shoot tips of numerous tropical and subtropical fruit tree species [67]. It is often referred to as ‘complete vitrification’ as vitrification takes place in both intra- and extracellular solutions [5]. This technique involves treatment (loading) of samples with a diluted mixture of cryoprotectors (LS) to elevate the explant’s resistance to more concentrated and toxic chemicals, dehydration with highly concentrated and efficient PVS, rapid cooling and rewarming, unloading (removing) cryoprotectants, and recovery [13]. The selection of a proper LS and its exposure time is important because of its impact on the dehydration tolerance of the tissues [68,69,70]. The most applicable LS consists of 2.0 M glycerol and 0.4 M sucrose. Studies on *Lilium japonicum* [71] and *Colocasia esculenta* [72] showed that incubation time in LS both longer and shorter than 20 min decreased the explants regeneration capacity. As for the plant vitrification solutions, PVS2 is the most popular one and consists of 30% (*w*/*v*) glycerol + 15% (*w*/*v*) DMSO + 15% (*w*/*v*) ethylene glycol (EG) + 0.4 M sucrose. The optimal exposure time to PVS2 varies between 15 and 25 min for shoot tips [73,74], although, 1–2 h incubation was effective in some orchid species [75]. Moreover, successful cryopreservation of tropical orchids was reported when the donor plants were precultured on a medium with a high concentration of sucrose for several days before vitrification and plunging in LN [76]. A study on around 20 tropical monocotyledonous plant species (e.g., banana, taro, and pineapple) revealed that cryopreservation of shoot tips through PVS2-based vitrification provided 100% shoot recovery if the mother plants were precultured on MS medium with 60–120 g·L^−1^ sucrose for 30 days before plunging in LN [76]. Conversely, PVS2-induced vitrification was lethal for the protocorms of *Paralophia epipyhtica*—a very rare orchid [77]. Other solutions, such as PVS3—50% glycerol (*w*/*v*) + 1.46 M sucrose, and PVS4—35% glycerol (*w*/*v*) + 20% (*w*/*v*) EG + 0.6 M sucrose, with no toxic DMSO, are also used with ornamental plant species [15,78]. “Toxicity” or “excessive dehydration effect” is the greatest problem of the concentrated vitrification solutions. This can sometimes be overcome by cold and osmotic hardening of plants and/or of the excised meristems and the application of PVS at 0 °C instead of at room temperature [5]. Another limitation is the very small size of the explants (1–4 mm), which can get easily injured during handling and transferring from one solution to another. Straw vitrification can be an interesting modification of the existing approach. In this protocol, rapid freezing rates are obtained by transferring the meristem into plastic straws together with the vitrification solution, followed by an LN immersion [5]. Future studies should focus on the utilization of this technique with ornamentals and fruit crops.

#### 4.2.6. Encapsulation-Vitrification

Encapsulation-vitrification, i.e., one of the so-called combined techniques (Figure 2e and Figure 3), is a combination of encapsulation-dehydration and vitrification techniques, in which explants are embedded in an alginate matrix and then treated with LS and PVS [13,79]. Due to the presence of the capsule, the incubation time in PVS has to be prolonged, though the dehydration process is more mild and safe for the samples [15]. The encapsulation-vitrification technique was efficiently used for *Lilium ledebourii* [61], *Saintpaulia ionantha* [72], *Dendrobium candidum* [80], *Grammatophyllum speciosum* [59], *Dendrobium nobile* [58], and an increasing number of tropical and subtropical fruit trees species [67]. Unfortunately, the combined techniques, especially with the use of DMSO (PVS2), are less popular with ornamental plants, because the long exposure to DMSO causes a breakdown of the alginate bead, and therefore, limited protection of the biological material. The application of these techniques could become more popular in the future, after optimizing the procedures [15].

#### 4.2.7. Droplet-Vitrification

Droplet-vitrification is a relatively new cryopreservation technique (Figure 2f,g and Figure 3), based on a method of cryopreserving cassava shoot tips (*Manihot esculenta*) in droplets of DMSO [6]. It can be considered the first “generic” cryopreservation method for hydrated tissues as it combines droplet-freezing and vitrification procedures [81]. The first report related to the PVS2-based droplet-vitrification method was presented by Pennycooke and Towill [82] on cryopreservation of sweet potato shoot tips. Panis et al. [36] optimized the droplet-vitrification procedure in a study with shoot tips of banana (*Musa* spp.). In the droplet-vitrification method, samples (incubated previously in LS and PVS) are placed in a drop of PVS (approximately 15 µL) on aluminum foil strips (approximately 5 × 20 mm) prior to direct plunging in LN. A detailed procedure of droplet-vitrification has been presented by Wang et al. [6]. The technique is based on the high cooling and warming rates (about 130 °C·s^−1^) obtained due to the good thermal conductivity of aluminum [15,36] and because of the direct contact between the explants and LN/unloading solution during cooling/rewarming, respectively [67]. In comparison, the cooling rates in a cryovial or artificial seed are about 6 °C·s^−1^ [83,84]. Other advantages of the droplet-vitrification method are as follows: avoiding the manipulation of the explant alone when inserting or extracting the foil strip from the cryovial (i.e., minimizing the risk of explant injury), placing the samples in droplets of vitrification solution already on the foil strips for dehydration and reducing the exposure time to PVS2 which is toxic to the cells, achieving vitrified state and avoiding devitrification during the cooling and rewarming, maintaining cell integrity of biological material and high regrowth percentage after LN-storage [6,15,36,85]. On the other hand, some of the most important problems of this cryopreservation technique include the requirement for a high level of technical skill, damage to or loss of samples due to the use of pipettes/tweezers for adding or removing solutions, and the need of precise control of the exposure time to PVS [6,36,86,87]. Droplet-vitrification was successfully applied for the cryopreservation of several endangered tropical and subtropical ornamental plants (Table 1) such as lilies, orchids, and redwood [59,88]. Moreover, this protocol has now been used with 1117 *Musa* accessions [6], 111 vegetable plant species such as potato and its wild relatives, *Diospyros*, cassava, yam, sweet potato, and some other tropical and subtropical fruit trees [13,39]. It is currently the most widely used storage technique for plant germplasm within cryobanks [5,6,79,89].

#### 4.2.8. Cryo-Plates and Cryo-Mesh

Recently, new cryogenic techniques using cryo-plates have been developed: V cryo-plate or vitrification cryo-plate (based on PVS2-vitrification of explants on a cryo-plate [90]) and D cryo-plate or dehydration cryo-plate (based on air dehydration [91]). In these techniques, explants, especially shoot tips, are placed on aluminum cryo-plates containing tiny wells [6]. The V cryo-plate technique combines encapsulation-vitrification with droplet-vitrification and the D cryo-plate combines alginate encapsulation with air drying (Figure 3). Cooling and warming rates in the cryo-plate methods are very high. Easy handling of samples kept in aluminum plates and high regrowth rates are two main advantages of those techniques [91]. However, the exposure time to PVS is longer in the V cryo-plate compared to droplet-vitrification, since the samples are encapsulated [90,91,92]. The D cryo-plate method may be used for larger explants and it is less laborious than the other cryostorage techniques. Moreover, this approach minimizes the risks of chemical stress, damage to biological material during manipulation, and possible genetic variation that could be induced by exposure to PVS [6,92]. The V and D cryo-plate methods have been used for cryopreservation of more than 25 and 15 tropical and subtropical ornamental and fruit plants, respectively [90,91,92,93,94,95,96,97,98,99], with comparable shoot regrowth percentages for both methods [91,96,97,98]. 

An alternative method for cryopreservation of shoot tips of tropical and subtropical ornamental and fruit plants may be a cryo-mesh. The main principles of the cryo-mesh cryopreservation method are similar to the V cryo-plate technique [90]. The major difference is that a stainless-steel mesh strip is used for cryo-mesh. Detailed procedures of cryo-mesh cryopreservation have been presented by Yamamoto et al. [90], Funnekotter et al. [100], and Wang et al. [6]. Another possibility is the user-friendly vitrification of tissues on electron microscope grids (made of copper) for cryopreservation, which so far has been successfully used with animal specimens (oocytes) [101].

#### 4.2.9. Dormant Bud Cryopreservation

Cryo-storage of dormant buds is a recent cryopreservation technique (Figure 3) [39]. Unfortunately, the number of species that can be stored via cryopreservation of dormant buds is limited as two main requirements must be met for this technique: (i) the species must produce buds that go into a dormant phase induced by a photoperiod and/or prolonged period of low temperature before being prepared for cryo-storage, and (ii) the LN-recovered buds must respond to bud grafting [39]. Dormant buds’ cryopreservation is based on the natural cold acclimatization of mother plants and controlled dehydration (by air dehydration and slow freezing) of scions holding dormant buds [6,102]. A significant advantage of this approach over the other cryopreservation techniques is that the in vitro culture phase is not involved during the whole procedure; the samples for conservation are transferred directly from the field to the LN tank and, at the time of recovery, back from the Dewar tank to the field [39]. This technique spares much time, resources, and reduces the risk of contamination [39,103]. Nonetheless, it applies to moderately to very cold-hardy woody species [67]. To date, only *Malus*, *Pyrus*, *Prunus*, and *Morus* have been cryopreserved with this technique [39,104]. The detailed dormant bud cryopreservation method has precisely been presented by Panis et al. [39].

**Table 1:** Cryopreservation of different tropical and subtropical ornamental plant species by various techniques.

Species and/or Cultivar	Explant Used	Method Applied	Survival (Recovery) [%]	Reference
*Bletilla striata*	Immature seeds	Direct immersion in LN vitrification	8 81–92	[105]
*Bletilla striata*	Mature seeds ^a^, Germinating seeds ^b^, Protocorms^c^	Droplet-vitrification	93 ^a^ 91 ^b^ 84 ^c^	[106]
*Brassidium* Shooting Star	PLBs	Droplet-vitrification	30	[85]
*Brassidium* Shooting Star	PLBs	Vitrification	No data	[107]
*Brassidium* Shooting Star	PLBs	Vitrification	No data	[108]
*Buxus hyrcana*	Shoot tips	Encapsulation-dehydration	60.00	[16]
*Buxus sempervirens*	Shoot tips	Encapsulation-dehydration ^a^ Encapsulation-vitrification ^b^	66.30 ^a^ 60.00 ^b^	[109]
*Cattleya* spp.	Seeds	Vitrification	No data	[110]
*Celisostoma areitinum*	Protocorms	Encapsulation-dehydration	49	[111]
*Centaurium rigualii*	Nodes	Encapsulation-dehydration	70	[112]
*Cymbidium hookerianum*	PLBs	Preculture	70	[113]
*Cyrtopodium hatschbachii*	Immature seeds	Encapsulation-dehydration	64	[114]
*Dendrobium cruentum*	Protocorms	Vitrification Encapsulation-dehydration	33 27	[115]
*Dendrobium candidum*	PLBs	Encapsulation-vitrification	85–89	[80]
*Dendrobium cariniferum*	Protocorms	Encapsulation-vitrification	15	[115]
*Dendrobium heterocarpum*	Protocorms	Encapsulation-dehydration	8	[116]
*Dendrobium nobile*	PLBs	Encapsulation-dehydration	53 (50)	[58]
*Dendrobium nobile*	PLBs	Encapsulation-vitrification	78 (76)	[58]
*Dendrobium* Walter Oumae	Shoot tips	Encapsulation-dehydration	16 (13)	[117]
*Doritis pulcherrima*	Seeds	Vitrification	62	[118]
*Fritillaria imperialis* Lubra Maxima	Bulb scale	Encapsulation-dehydration ^a^ Encapsulation-vitrification ^b^	74.30 ^a^ 81.6 ^b^	[119]
*Gentiana cruciata*	Proembryogenic masses	Droplet-vitrification	82	[120]
*Gentiana cruciata*	Proembryogenic masses	Slow cooling	2.5–2.7	[121]
*Gentiana cruciata*	Proembryogenic masses	Vitrification	86–91	[121]
*Gentiana scabra*	Axillary buds	Preculture-desiccation	5–90	[49]
*Gentiana* sp.	Shoot tips ^a^ Axillary buds ^b^	Vitrification	74 ^a^ 78 ^b^	[79] ^a^, [49] ^b^
*Grammatophyllum speciosum*	Protocorms	Droplet-vitrification	38	[59]
*Grammatophyllum speciosum*	Protocorms	Encapsulation-dehydration	24	[59]
*Grammatophyllum speciosum*	Protocorms	Encapsulation-vitrification	14	[59]
*Lilium japonicum*	Apical meristems	Vitrification	68	[71]
*Lilium* sp.	Shoot tips	Vitrification	60–90	[122]
*Lilium* spp.	Shoot tips	Droplet-vitrification	42–87	[123]
*Lilium ledebourii*	Seeds ^a,c,d,e^ Embryogenic axes ^a,b,c^ Lateral buds ^a,c^ Bulblet ^a,c^ Shoot tips ^f^	Direct immersion in LN ^a^/Vitrification ^b^/Encapsulation-vitrification^c^/Encapsulation-dehydration ^d^/Preculture-desiccation ^e^ Droplet-vitrification ^f^	0 ^a^/10 ^c^/50 ^d^/75 ^e^ 0 ^a/b^/10 ^c^ 0 ^a/c^ 0 ^a/c^ 58–90 (53–88) ^f^	[48,60,61] ^a–e^, [124] ^f^
*Lilium*× *siberia*	Apical meristems	Vitrification/Droplet-vitrification	35–45/35–84	[88]
*Magnolia macrophylla*	Shoot tips	Droplet-vitrification	30	[125]
*Magnolia sinica*	Seeds	Desiccation	58	[126]
*Magnolia sirindhorniae*	Shoot tips	Encapsulation-vitrification	33	[127]
*Oncidium* sp.	PLBs	Preculture-desiccation	30	[128]
*Oncidium bifolium*	Seeds Protocorms	Encapsulation-dehydration	67 (5) 82 (11)	[129]
*Oncidium flexuosum*	Seeds	Vitrification	78	[130]
*Phaius tankervillae*	Seeds	Vitrification	62	[131]
*Phalaenopsis bellina*	PLBs	Encapsulation-dehydration	47	[64]
		Preculture-desiccation	30	[64]
*Pinus nigra*	Proembryogenic masses	Slow cooling	88	[132]
*Rhynchostylis gigantean*	Protocorms	Vitrification	19	[115]
*Rosa* × *hybrida*	Shoot tips Axillary buds	Droplet-vitrification ^a^ Encapsulation-dehydration ^b^	(58–64) ^a^ (12) ^a^ (0) ^b^	[133] ^a^, [134] ^b^
*Seidenfadenia mitrata*	Protocorms	Vitrification	67	[115]
*Vanda coerulea*	PLBs	Droplet-vitrification	5	[135]
*Vanda coerulea*	Protocorms	Encapsulation-dehydration	40	[136]
*Vanda coerulea*	Seeds	Vitrification	67	[137]
*Vanda tricolor*	Mature seeds	Direct immersion in LN vitrification	10 14	[68]
*Vanilla planifolia* Andrews	Shoot apices	Droplet-vitrification	30 (10)	[138]

Upper lowercase letter refers to the explant type, the cryopreservation technique used, and its effectiveness.

**Table 2:** Cryopreservation of different fruit plant species by various techniques.

Plant Species	Explant	Cryopreservation Technique	Survival (%)	Reference
*Actinidia* spp.	Shoot tips	Droplet-vitrification	59–88	[139]
		Encapsulation-dehydration	85–95	[140]
*Agave peacockii*	Shoot tips	Droplet-vitrification	96	[141]
*Agave tequilana*	Somatic embryos	V cryo-plate technique	83	[142]
*Ananas* spp.	Shoot tips	Droplet-vitrification	51	[143]
*Ananas* (wild genus)	Pollen	Dehydration	62	[144]
*Citrus sinensis*	Callus	Modified aluminum cryo-plate	88	[145]
*Citrus* spp.	Shoot tips	Droplet-vitrification	56	[146]
*Cocos nucifera*	Shoot tips	Droplet-vitrification	50	[147]
*Diospyros kaki*	Shoot tips	D cryo-plate	67–97	[99]
*Diospyros kaki*	Shoot tips	Encapsulation-droplet-vitrification ^a^ Slow freezing ^b^ Vitrification ^c^	80 ^a^ 70–76 ^b^ 86 ^c^	[148] ^a^, [149] ^b^, [150] ^b^, [151] ^c^
*Diospyros* spp.	Shoot tips	Vitrification ^a^	30 ^a^ 100 ^b^	[152] ^a^, [153] ^b^
*Musa* spp.	Apical meristems	Droplet-vitrification	39	[36]
*Musa* spp.	Cell suspensions	Classical (slow) freezing	No data	[154]
*Passiflora edulis*	Zygotic embryos	Dehydration	100	[155]
*Passiflora suberosa*	Shoot tips	Encapsulation-vitrification	28	[156]
*Passiflora pohlii*	Nodal segments	Vitrification	65	[157]
*Persea americana*	Shoot tips	Vitrification	73–80	[158]
*Persea americana*	Somatic embryos	Cryovial-vitrification ^a^ Droplet-vitrification ^b^	73–91 ^a^ 85–100 ^b^	[159]
*Prunus cerasifera*	Shoot tips	V and D cryo-plates	56.1% (V cryo-plate) and 77.5% (D cryo-plate)	[97]
*Prunus cerasus*	Dormant bud	No data	No data	[160]
*Prunus domestica*	Shoot tips	V and D cryo-plates	44.6% (V cryo-plate) and 47.5% (D cryo-plate)	[97]
*Prunus* spp.	Shoot tips	Encapsulation-dehydration ^a^ Vitrification ^b^ Droplet-vitrification ^c^ Two-step freezing ^d^	14–76 ^a^ 60–88 ^b^ 20–52 ^c^ 74 ^d^	[161] ^a^, [162] ^b^, [97] ^c^, [163] ^c^, [163] ^d^
*Pyrus* spp.	Shoot tips	Encapsulation-dehydration ^a^ Vitrification ^b^ Two step freezing ^c^	30–82 ^a^ 71 ^b^ 75–83 ^c^	[164] ^a^, [165] ^b^, [166] ^c^, [167] ^c^

Upper lowercase letter refers to the cryopreservation technique used and its effectiveness.

## 5. Rewarming and Recovery

Optimized rewarming is vital to prevent glass relaxation and devitrification. Ice recrystallization during warming, i.e., merging smaller crystals into larger aggregates, would result in cell death. Consequently, rewarming ought to be done rapidly, either by transferring the vials with samples to a 35–42 °C (usually 38 °C) water bath for 2–3 min or by plunging the aluminum foils containing explants in the unloading solution at room temperature for better *post*-storage recovery [19]. Occasionally, a two-phase approach can be used: first, a short phase (i.e., 1–2 min at ambient temperature) to allow glass relaxation, proceeded by rapid warming (at +45 °C) to guarantee the rapid transition from biological glass to liquid without passing through an ice phase [19,32]. 

After rewarming, the used solutions (e.g., PVS) or materials (e.g., capsule) may be removed before the recovery step (Figure 2h). The composition of the recovery medium (RM) is a vital factor affecting the success of a cryopreservation protocol [15]. Rehydration takes place about a few hours after explant inoculation on the RM. In order to enhance the penetration of nutrients, it is advised to inoculate the samples on a semi-solid or sometimes liquid media [15]. 

One of the drawbacks of cryopreservation is the problem with complete plant recovery. It is sometimes observed that, despite the high survival rate of the LN-stored explants, their further development is arrested [1]. Therefore, the suitable combination of plant growth regulators (PGRs) in RM, particularly an auxin and cytokinin, may be crucial to stimulate the regeneration of LN-derived tissues [15] and to proceed with direct organogenesis and embryogenesis, although callus formation should be avoided due to the risk of somaclonal variation occurrence that is unacceptable in the long-term storage of germplasm. Moreover, exogenous applications of enzymatic and non-enzymatic antioxidants, such as catalase (CAT), pyruvate dehydrogenase (PDH), malate dehydrogenase (MDH), or melatonin can be used to alleviate oxidative stress for improving plant cryopreservation efficacy [168].

## 6. Cryopreservation and Omics Technologies

The word omics refers to an area of biology studies including genomics, proteomics, or metabolomics. The ending “-ome” is used to highlight the study object of such areas, i.e., the genome, proteome, or metabolome, respectively. Omics science aims to recognize, describe, and quantify all the biological molecules involved in the structure, function, and dynamics of a cell, tissue, organ, or organism. Biomics or bionomics is a biological science focused on organisms’ habitats and modes of life in their natural environment. Cryobiomics or cryobionomics, on the other hand, is a branch of biology dealing with cryopreserved organisms’ behavior, habitats, stability, and function following their reintroduction into the natural environment [31]. Cryobiomics connects causal factors related to cryoinjury and loss of viability to the risks of genetic instability [31]. In other words, cryobiomics is a relationship between cryoinjury and the (epi)genetic integrity of cryopreserved plant cells, as well the potential impact of cryoinjury on the genome, transcriptome, proteome, and metabolome [21]. The general ontological term of omics describes the application of (functional) genomics, and bioinformatics as generating an abundance of DNA sequence data that reveal the complexity of global changes in the expression of genes, additionally supported by profiling through powerful transcriptomics techniques [31]. The array of global changes in proteins via proteomics is a fundamental bridge between the transcriptome and metabolome, with metabolomics providing a global profile of a wide range of metabolites and cell signaling processes. Bioinformatics and gene ontology are central tools that unify omics platforms across biology [169,170]. The implications of cryobiomics have been considered in a number of applications, from algal culture to tropical and subtropical plant biodiversity conservation [40,171].

Plant cryopreservation is related to biomolecular and omics sciences through the understanding of the stability of the stored sample [35,172], as some theoretical and empirical data suggests that molecular, physiological, and biochemical processes may not be entirely stopped at ultra-low temperatures [173]. Cryopreservation results in the exposure of cells to chemical, physical, and physiological stresses. Cryoinjury caused by freezing/thawing (in slow-cooling) and cooling/rewarming (in vitrification-based modern techniques) may influence the DNA, cell membrane structure and function, molecular and subcellular functions, totipotency, and finally regrowth of entire plants and their field performance [174]. Some of the most important problems when exposing cells to cryopreservation include osmotic injury during dehydration, hyperhydration during recovery, toxicity of cryoprotective agents/mixtures, oxidative damage induced by reactive oxygen species (ROS), and secondary lipid peroxidation aldehyde products [35,175,176]. The perception of stress by cells and the linked cascades provoked by signaling molecules activate the expression of transcription factors (TF) that impact numerous stress response genes [172]. Calcium may be a messenger in the signal transduction process that occurs during cold acclimatization and cryopreservation, which cause a complex series of pathways revealed by mutant, (functional) genomics, proteomics, transcriptomics, and metabolomics studies [32]. ABA also has a significant role in preventing osmotic stress caused by dehydration prior to cryopreservation [172,177]. Cold acclimation provokes an intrinsic tolerance to desiccation and low temperature by triggering genes related to cold adaptation [178,179]. Through these processes, biosynthesis of ethylene, changes in ROS production (such as the superoxide anion, hydrogen peroxide, and hydroxyl radicals), as well as membrane fluidity may be induced [175,180]. Antioxidant mechanisms can also positively affect the *post*-storage viability of cells [175,181]. The utilization of carbohydrates, on the other hand, by osmoprotection of the cell membrane (through the interaction with lipid bilayer) promotes its resistance to the toxic effects of PVS and desiccation [88,133,182]. Moreover, the activity of carbohydrates, especially sucrose in the encapsulation-based protocols, results in a reduction in the melting temperature of ice during rewarming [183]. 

By utilizing high-throughput omics technology to screen two dehydrins of embryogenic callus of *Agapanthus praecox* subjected to cryopreservation, Yang et al. [184] reported that their expression levels were specifically upregulated at the transcription and protein levels. Chen et al. [185], on the other hand, markedly increased the recovery level of cryopreserved embryogenic cells in this species and enhanced the expressions of stress-responsive genes, including POD, APX, MDHAR, and GPX through the inclusion of 0.08 mM glutathione in PVS2. In the study with *Dendrobium nobile*, Di et al. [186] reported that protein synthesis, processing, and degradation might be the main strategies to re-establish cell balance in the PLBs following LN-storage. In cryopreservation of *Dendrobium* PLBs by vitrification, Jiang et al. [187] reported that the preculture and cooling–rewarming cycle induced expression of the autophagy-related protein 8C gene (*Atg 8C*) and reticulon-like protein B8 gene (*Rtnl B8*). These results provided evidence on ROS-triggered programmed cell death during cryopreservation. A series of enzymatic and ROS analyses in several orchid species and *Passifora suberosa* allowed to develop robust cryopreservation protocols by sufficient maintenance of the internal balance of oxidative metabolism [188,189,190,191].

In cryobiomics, it is desirable to determine the genetic integrity, gene expression, as well as growth and development of the cryopreserved plants to evaluate the possible cellular and/or biochemical damage, impairment of metabolism, loss of reproductive functions, and, ultimately, to assess if they are true-to-type. This can be performed at the phenotypic, cytological, histological, physiological, genetic, epigenetic, and molecular levels [21,40]. Another perspective of cryobiomics is that molecular alternations, especially at the epigenetic level, may be indicative of a beneficial adaptive response to the stresses incurred during LN-storage and which can be advantageous to *post*-storage survival [192]. Genomics and molecular markers (e.g., amplified fragment length polymorphism (AFLP), inter simple sequence repeat (ISSR), random amplified polymorphic DNA (RAPD), and simple sequence repeat (SSR)) have an important role in cryobiomics [6,92]. Previous studies have shown no or small genetic differences between cryopreserved and non-cryopreserved samples under optimized conditions [193,194,195]. However, more sensitive and powerful tools, such as whole-genome bisulfite sequencing (WGBS) and methylation-sensitive amplified polymorphism (MSAP), may be applied to validate more comprehensively the (epi)genetic homogeneity in the germplasms recovered from LN [6,25,92,195]. Histone acetylation, small interfering RNA (siRNA), microRNAs, and the role of the association between somaclonal variation and DNA methylation, are new candidate markers for variation detection [31]. Likewise, the combination of genetic engineering and cryopreservation techniques can be helpful to study genes involved in the tolerance to dehydration and low temperatures [19].

## 7. Conclusions and Future Perspectives

Currently, about 22,000 plant species and cultivars are on the red list of the International Union for Conservation of Nature and Natural Resources, including extinct, extinct in the wild, critically endangered, endangered, vulnerable, and near-threatened species [196]. Advances in plant biotechnology improve the long-term conservation and management of biodiversity. Cryopreservation may play a central role in the safe storage of important genetic resources of tropical and subtropical ornamental and fruit crops. Nonetheless, additional research on recalcitrant species of tropical and subtropical fruit and ornamental plants is needed. Droplet-vitrification and cryo-plate techniques have the mutual characteristic of providing higher cooling and warming rates than the other vitrification-based methods because explants are placed on aluminum foil strips or cryo-plates (with a very high thermal conductivity) and are in direct contact with LN during cooling and with the unloading solution during rewarming. 

Obviously, cryopreservation is a complimentary method to other in situ and ex situ conservation strategies and has certain drawbacks. Among the demerits of plant cryopreservation one can mention: it does not work efficiently with all plant material, hence cryostorage protocols for many plant species are not available; ice crystallization inside the cells cause injury to the organelles, whereas cellular dehydration can induce stress; high intracellular concentration of solutes can be very damaging to cells but also cryoprotectants affect the viability of cells; successful cryogenics currently mostly succeed for very small organs and structures, and this is still a constraint; finally, the physiological status of the donor plant material is of high importance.

Nowadays, the use of high-throughput omics technologies aids to identify functions of the specific genes and proteins in protecting the cells against cryopreservation-induced stress. Future research may lead toward the: improvement in *post*-LN-storage recovery of plants; metabolic, genetic, and epigenetic stability; survey of cryopreserved-mediated genes; detection of precise molecular markers; improvement in combined techniques (especially encapsulation-vitrification, cryo-plates, cryo-mesh, and straw vitrification); the use of SD-BAB, SLS-BABs, microtubers, dormant buds, and rhizome buds as alternative explants in cryopreservation; better adaptation of cryo-derived plants to native conditions; improving the tolerance of explants to dehydration, vitrification, and cold stress; introduction of newer PVS, and supplementation of different types of antioxidants; optimizing universal protocols for a wider group of plants; understanding the different protective mechanisms and stress conditions involved in cryostorage; development of protocols for pathogen eradication by means of cryotherapy; and extending the current status of long-term conservation for economically important plant species that fall out of the ‘model system’ framework.

## Data Availability

Not applicable.

## References

[1] Roque-Borda C.A., Kulus D., de Souza A.V., Kaviani B., Vicente E.F. (2021). Cryopreservation of agronomic plant germplasm using vitrification-based methods: An overview of selected case studies. Int. J. Mol. Sci..

[2] Krupnick G.A. (2013). Conservation of tropical plant biodiversity: What have we done, where are we going?. BioTropica.

[3] Corlett R.T. (2020). Safeguarding our future by protecting biodiversity. Plant Diver..

[4] Reed B.M. (2001). Implementing cryopreservation storage of clonally propagated plants. Cryo-Letters.

[5] Panis B. (2019). Sixty years of plant cryopreservation: From freezing hardy mulberry twigs to establishing reference crop collections for future generations. Acta Hortic..

[6] Wang M.R., Lambardi M., Engelmann F., Pathirana R., Panis B., Volk G.M., Wang Q.C. (2020). Advances in cryopreservation of in vitro-derived propagules: Technologies and explant sources. Plant Cell Tissue Org. Cult..

[7] Bernard F., Shaker-Bazarnov H., Kaviani B. (2002). Effect of salicylic acid on cold preservation and cryopreservation of encapsulated embryonic axes of Persian lilac (*Melia azedarach* L.). Euphytica.

[8] Engelmann F. (2011). Use of biotechnologies for the conservation of plant biodiversity. In Vitro Cell. Dev. Biol. Plant.

[9] Li J.-W., Zhang X.-C., Wang M.-R., Bi W.-L., Faisal M., da Teixeira Silva J.A., Volk J.M., Wang Q.-C. (2019). Development, progress and future prospects in cryobiotechnology of *Lilium* spp.. Plant Methods.

[10] Engelmann F. (2004). Plant cryopreservation: Progress and prospects. In Vitro Cell. Dev. Biol. Plant.

[11] Panis B., Piette B., Andreé E., Van den Houwe I., Swennen R. (2011). Droplet vitrification: The first generic cryopreservation protocol for organized plant tissues?. Acta Hortic..

[12] Kaviani B. (2011). Conservation of plant genetic resources by cryopreservation. Aust. J. Crop Sci..

[13] Cruz-Cruz C.A., González-Arnao M.T., Engelmann F. (2013). Biotechnology and conservation of plant biodiversity. Resources.

[14] Acker J.P., Adkins S., Alves A., Horna D., Toll J. (2017). Feasibility study for a safety back-up cryopreservation facility. Independent Expert Report.

[15] Kulus D., Zalewska M. (2014). Cryopreservation as a tool used in long-term storage of ornamental species—A review. Sci. Hortic..

[16] Kaviani B., Negahdar N. (2017). Propagation, micropropagation and cryopreservation of *Buxus sempervirens* Pojark., an endangered ornamental shrub. S. Afr. J. Bot..

[17] Fukai S. (1989). Plant regeneration from shoot tips of *Dianthus* hybrid cryopreserved in liquid nitrogen up to 2 years. Plant Tissue Cult. Lett..

[18] Sakai A. (1956). Survival of plant tissue of super-low temperature. Contrib. Inst. Temp. Sci. Haikkaido Univ. Ser. B.

[19] González-Arnao M.T., Martinez-Montero M.E., Cruz-Cruz C.A., Engelmann F., Ahuja M.R., Ramawat K.G. (2014). Advances in cryogenic techniques for the long-term preservation of plant biodiversity Maria Teresa. Biotechnology and Biodiversity, Sustainable Development and Biodiversity.

[20] Wang B., Zhang Z., Yin Z., Feng C., Wang Q. (2012). Novel and potential application of cryopreservation to plant genetic transformation. Biotechnol. Adv..

[21] Harding K. (2004). Genetic integrity of cryopreservaed plant cells: A review. CryoLetters.

[22] Martinez-Montero M.E., Harding K., Barh D., Khan M., Davies E. (2015). Cryobionomics: Evaluating the concept in plant cryopreservation. Plant Omics: The Omics of Plant Science.

[23] Gabellini S., Scaramuzzi S. (2022). Evolving consumption trends, marketing strategies, and governance settings in ornamental horticulture: A grey literature review. Horticulturae.

[24] Salachna P. (2022). Trends in ornamental plant production. Horticulturae.

[25] Normah M.N., Sulong N., Reed B.M. (2019). Cryopreservation of shoot tips of recalcitrant and tropical species: Advances and strategies. Cryobiology.

[26] Malik S.K., Chaudhury R., Rajasekharan P., Rao V. (2019). Cryopreservation techniques for conservation of tropical horticultural species using various explants. Conservation and Utilization of Horticultural Genetic Resources.

[27] Viracheva L.L., Goncharova O.A., Kirillova N.R., Nosatenko O.Y., Trostenyuk N.N. (2019). Rare and disappearing plants in the introductive collection of the Polar-Alpine Botanical Garden and Institute. Hort. Bot..

[28] Global Fruit Production in 2020. www.statista.com.

[29] Zamecnik J., Faltus M., Bilavcik A. (2021). Vitrification solutions for plant cryopreservation: Modification and properties. Plants.

[30] Kulus D., Abratowska A., Mikuła A. (2018). Morphogenetic response of shoot tips to cryopreservation by encapsulation-dehydration in a solid mutant and periclinal chimeras of *Chrysanthemum × grandiflorum*/Ramat./Kitam. Acta Physiol. Plant.

[31] Barh D., Sarwar Khan M., Davies E. (2015). Plant Omics: The Omics of Plant Science.

[32] Benson E.E. (2008). Cryopreservation of phytodiversity: A critical appraisal of theory & practice. Crit. Rev. Plant Sci..

[33] Uemura M., Minami A., Kawamura Y. (2009). Effect of low temperature and cryoprotectants on plant plasma membrane. 1st International Symposium: Cryopreservation in Horticultural Species, Book of Abstracts.

[34] Ciani F., Cocchia N., Esposito L., Avallone L., Wu B. (2012). Fertility cryopreservation. Advances in Embryo Transfer.

[35] Benson E.E., Betsou F., Fuller B.J., Harding K., Kofanova O. (2013). Translating cryobiology principles into transdisciplinary storage guidelines for biorepositories and biobanks: A concept paper. CryoLetters.

[36] Panis B., Piette B., Swennen R. (2005). Droplet vitrification of apical meristems: A cryopreservation protocol applicable to all Musaceae. Plant Sci..

[37] Panis B., Lambardi M. (2005). Status of cryopreservation technologies in plants (crops and forest trees). The Role of Biotechnology.

[38] Jiroutová P., Sedlák J. (2020). Cryobiotechnology of plants: A hot topic not only for gene banks. Appl. Sci..

[39] Panis B., Nagel M., Van den Houwe I. (2020). Challenges and prospects for the conservation of crop genetic resources in field genebanks, in in vitro collections and/or in liquid nitrogen. Plants.

[40] Benson E., Harding K., Debouck D., Dumet D., Escobar R., Mafla G., Panis B., Panta A., Tay D., Van den Houwe I. (2011). Refinement and standardization of storage procedures for clonal Crops-Global Public Goods Phase 2: Part II. Status of In Vitro Conservation Technologies for: Andean Root and Tuber Crops, Cassava, Musa, Potato, Sweet Potato and Yam.

[41] Engelmann F., Altman A., Hazegawa P.M. (2012). Germplasm collection, storage and preservation. Plant Biotechnology and Agriculture—Prospects for the 21st Century.

[42] González-Arnao M.T., Panta A., Roca W.M., Escobar R.H., Engelmann F. (2008). Development and large scale application of cryopreservation techniques for shoot and somatic embryo cultures of tropical crops. Plant Cell Tissue Org. Cult..

[43] Benson E.E., Johnston J., Muthusamy J., Harding K., Gupta S., Ibaraki Y. (2006). Physical and engineering perspectives of in vitro plant cryopreservation. Plant Tissue Culture Engineering.

[44] Heine-Dobbernack E., Seufert S., Schumacher H.M. (2006). Controlled rate freezing of dedifferentiated plant cell lines—A mini-test system for quick evaluation of parameters. Cryobiology.

[45] Reed B.M., Reed B.M. (2008). Cryopreservation-practical considerations. Plant Cryopreservation: A Practical Guide.

[46] Engelmann F., Takagi H. (2000). Cryopreservation of Tropical Plant Germplasm: Current Research Progress and Application.

[47] Hamilton K.N., Ashmore S.E., Pritchard H.W. (2009). Thermal analysis and cryopreservation of seeds of Australian Wild *Citrus* species (Rutaceae): *Citrus australasica*, *C. inodora* and *C. garrawayi*. CryoLetters.

[48] Kaviani B., Abadi D.H., Torkashvand A.M., Hoor S.S. (2009). Cryopreservation of seeds of lily (*Lilium ledebourii* Baker Bioss.): Use of sucrose and dehydration. Afr. J. Biotechnol..

[49] Suzuki M., Ishikawa M., Okuda H., Noda K., Kishimoto T., Nakamura T., Ogiwara I., Shimura I., Akihama T. (2006). Physiological changes in Gentian axillary buds during two-step preculturing with sucrose that conferred high levels of tolerance to desiccation and cryopreservation. Ann. Bot..

[50] Engelmann F., Callow J.A., Ford-Lloyd B.V., Newbury H.J. (1997). In vitro conservation methods. Biotechnology and Plant Genetic Resources.

[51] Wilkinson T., Wetten A., Prychid C., Fay M.F. (2003). Suitability of cryopreservation for the long-term storage of rare and endangered plant species: A case history of *Cosmos atrosanguineus*. Ann. Bot..

[52] González-Arnao M.T., Lazaro-Vallejo C.E., Valencia M.G., Ortiz Patraca N.M., Tex-cahua Martinez H. (2010). Adjustment of cryoprotective conditions for vanilla (*Vanilla planifolia*) shoot-tips subjected to a droplet-vitrification protocol. Cryobiology.

[53] Engelmann F. (2009). Encapsulation-dehydration: Past, present and future. Acta Hortic..

[54] da Teixeira Silva J.A., Zeng S., Galdiano R.F., Dobránszki J., Cardoso J.C., Vendrame W.A. (2014). In vitro conservation of *Dendrobium* germplasm. Plant Cell Rep..

[55] Ozden-Tokatli Y., De Carlo A., Gumusel1 F., Pignattelli S., Lambardi M. (2008). Development of encapsulation techniques for the production and conservation of synthetic seeds in ornamental species. Propag. Ornam. Plants.

[56] Kaviani B., Bernard F., Shakeri H., Hadadchi G.H.R. (2005). Effects of salic acid on enhancing the resistance of embryonic axes of Persian lilac (*Melia azedarach* L.) against cold and cryopreservation. Agron. Hortic..

[57] Kaviani B. (2007). Effects of salicylic acid and encapsulation on enhancing the resistance of embryonic axes of Persian lilac (*Melia azedarach* L.) against cryopreservation. Int. J. Agri. Biol..

[58] Mohanty P., Das M.C., Kumaria S., Tandon P. (2012). High-efficiency cryopreservation of the medicinal orchid *Dendrobium nobile* Lindl. Plant Cell Tissue Org. Cult..

[59] Sopalun K., Kanchit K., Ishikawa K. (2010). Vitrification-based cryopreservation of *Grammatophyllum speciosum* protocorm. CryoLetters.

[60] Kaviani B., Safari-Motlagh M.R., Padasht-Dehkaei M.N., Darabi A.H., Rafizadeh A. (2008). Cryopreservation of lily [*Lilium ledebourii* (Baker) Bioss.] germplasm by encapsulation-dehydration. Int. J. Bot..

[61] Kaviani B., Dahkaei M., Hashemabadi D., Darabi A. (2010). Cryopreservation of *Lilium ledebourii* (Baker) Bioss. by encapsulation-vitrification and in vivo media for planting of germplasm. Am.-Eur. J. Agric. Environ. Sci..

[62] Kaviani B. (2010). Cryopreservation by encapsulation-dehydration for long-term storage of some important germplasm: Seed of lily [*Lilium ledebourii* (Baker) Bioss.], embryonic axe of Persian lilac (*Melia azedarach* L.), and tea (*Camellia sinensis* L.). Plant Omics J..

[63] Yin L.L., Poobathy R., James J., Julkifle A.L., Subramaniam S. (2011). Preliminary investigation of cryopreservation by encapsulation-dehydration technique on *Brassidium* Shooting Star orchid hybrid. Afr. J. Biotechnol..

[64] Khoddamzadeh A.A., Sinniah U.R., Lynch P., Kadir M.A., Kadzimin S.B., Mahmood M. (2011). Cryopreservation of protocorm-like bodies (PLBs) of *Phalaenopsis bellina* (Rchb. f.) Christenson by encapsulation-dehydration. Plant Cell Tissue Org. Cult..

[65] Subramaniam S., Sinniah U.R., Khoddamzadeh A.L., Periasamy S., James J.J. (2011). Fundamental concept of cryopreservation using *Dendrobium* Sonia-17 protocorm-like bodies by encapsulation-dehydration technique. Afr. J. Biotechnol..

[66] Bonnart R., Volk G.M. (2010). Increased efficiency using the encapsulation-dehydration cryopreservation technique for *Arabidopsis thaliana*. CryoLetters.

[67] Benelli C., De Carlo A., Engelmann F. (2013). Recent advances in the cryopreservation of shoot-derived germplasm of economically important fruit trees of *Actinidia*, *Diospyros*, *Malus*, *Olea*, *Prunus*. Biotechnol. Adv..

[68] Jitsopakul N., Thammasiri K., Yukawa C., Ishikawa K. (2012). Effect of cryopreservation on seed germination and protocorm development of *Vanda tricolor*. Sci. Asia..

[69] Sekizawa K., Yamamoto S., Rafique T., Fukui K., Niino T. (2011). Cryopreservation of in vitro-grown shoot tips of carnation (*Dianthus caryophyllus* L.) by vitrification method using aluminum cryo-plates. Plant Biotechnol..

[70] Kim H.-H., Popova E., No N.-Y., Back H.-J., Kim C.-K., Cho E.-G., Engelmann F. (2011). Application of alternative loading solutions to garlic and chrysanthemum in droplet-vitrification procedures. Acta Hortic..

[71] Matsumoto T., Sakai A., Yamada K. (1995). Cryopreservation of *in vitro*-grown apical meristems of lily by vitrification. Plant Cell Tissue Org. Cult..

[72] Moges A.D., Shibli R.A., Karam N.S. (2004). Cryopreservation of African violet (*Saintpaulia ionantha* Wendl.) shoot tips. In Vitro Cell. Dev. Biol. Plant.

[73] Takagi H., Tien Thinh N.T., Islam O.M., Senboku T., Sakai A. (1997). Cryopreservation of *in vitro*-grown shoot tips of taro (*Colocasia esculenta* (L.) Schott) by vitrification. 1. Investigation of basic conditions of the vitrification procedure. Plant Cell Rep..

[74] Thinh N.T., Takagi H., Towill L.E., Bajaj Y.P.S. (2002). Cryopreservation of *Colocasia esculenta* L. Schott (Taro). Biotechnology in Agriculture and Forestry.

[75] Antony J.J.J., Keng C., Rathinam X., Marimuthu S., Subramaniam S. (2011). Effect of preculture and PVS2 incubation conditions followed by histological analysis in the cryopreservaed PLBs of *Dendrobium* Bobby Messina orchid. Aust. J. Crop Sci..

[76] Thinh N.T. (1997). Cryopreservation of Germplasm of Vegetatively Propagated Tropical Monocots by Vitrification. Ph.D. Thesis.

[77] Cripps R.F., McGregor K. (2009). Determination of the optimal dehydration period for the protocorms of *Paralophia epipyhtica* (Orchidaceae) using differential scanning calorimetry. Cryobiology.

[78] Kim H.H., Lee Y.G., Shin D.J., Ko H.C., Gwag J.G., Cho E.G., Engelmann F. (2009). Development of alternative plant vitrification solutions in droplet-vitrification procedures. Cryobiology.

[79] Sakai A., Engelmann F. (2007). Vitrification, encapsulation-vitrification and droplet-vitrification: A review. CryoLetters.

[80] Yin M., Hong S. (2009). Cryopreservation of *Dendrobium candidum* Wall. ex Lindl. protocorm-like bodies by encapsulation-vitrification. Plant Cell Tissue Org. Cult..

[81] Sakai A., Kobayashi S., Oiyama I. (1990). Cryopreservation of nucellar cells of navel orange (*Citrus sinensis* Osb. var. *brasiliensis* Tanaka) by vitrification. Plant Cell Rep..

[82] Pennycooke J.C., Towill L.E. (2000). Cryopreservation of shoot tips from in vitro plants of sweet potato [*Ipomoea batatas* (L.) Lam.] by vitrification. Plant Cell Rep..

[83] Niino T., Yamamoto S., Matsumoto T., Engelmann F., Arizaga M.V., Tanaka D. (2019). Development of V and D cryo-plate methods as effective protocols for cryobanking. Acta Hortic..

[84] Towill L.E., Bonnart R. (2003). Cracking in a vitrification solution during cooling or warming does not affect growth of cryopreserved mint shoot tips. CryoLetters.

[85] Rahmah R., Mubbarakh S.A., Sinniah U.R., Subramaniam S. (2015). Effects of droplet-vitrification on *Brassidium* Shooting Star’s orchid protocorm-like bodies (PLBs). Sci. Hortic..

[86] Kim H.M., Shin J.H., Sohn J.K. (2006). Cryopreservation of somatic embryos of the herbaceous peony (*Paeonia lactiflora* Pall.) by air drying. Cryobiology.

[87] Sant R., Panis B., Taylor M., Tyagi A. (2008). Cryopreservation of shoot tips by droplet vitrification applicable to all taro (*Colocasia esculenta* var. *esculenta*) accessions. Plant Cell Tissue Org. Cult..

[88] Chen X.L., Li J.H., Xin X., Zhang Z.E., Xin P.P., Lu X.X. (2011). Cryopreservation of *in vitro*-grown apical meristems of *Lilium* by droplet-vitrification. S. Afr. J. Bot..

[89] Wang M.R., Chen L., da Teixeira Silva J.A., Volk G.M., Wang Q.-C. (2018). Cryobiotechnology of apple (*Malus* spp.): Development, progress and future prospects. Plant Cell Rep..

[90] Yamamoto S., Fukui K., Niino T. (2011). A new cryopreservation method for vegetatively propagated plant genetic resources using aluminum cryo-plates. Dev. Technol..

[91] Niino T., Yamamoto S., Fukui K., Martínez C.R.C., Arizaga M.V., Matsumoto T., Engelmann F. (2013). Dehydration improves cryopreservation of mat rush (*Juncus decipiens* Nakai) basal stem buds on cryo-plates. CryoLetters.

[92] Matsumoto T. (2017). Cryopreservation of plant genetic resources: Conventional and new methods. Rev. Agric. Sci..

[93] Salma M., Fki L., Engelmann-Sylvestre I., Niino T., Engelmann F. (2014). Comparison of droplet-vitrification and D-cryoplate for cryopreservation of date palm (*Phoenix dactylifera* L.) polyembryonic masses. Sci. Hortic..

[94] Matsumoto T., Yamamoto S., Fukui K., Niino T. (2014). Cryopreservation of blueberry dormant shoot tips using V cryoplate method. Ann. Conf. Am. Soc. Hortic. Sci..

[95] Rafique T., Yamamoto S., Fukui K., Mahmood Z., Niino T. (2015). Cryopreservation of sugarcane using the V cryo-plate technique. CryoLetters.

[96] Engelmann-Sylvestre I., Engelmann F. (2015). Cryopreservation of *in vitro*-grown shoot tips of *Clinopodium odorum* using aluminum cryo-plates. In Vitro Cell. Dev. Biol. Plant.

[97] Vujović T., Chatelet P., Ružića D., Engelmann F. (2015). Cryopreservation of *Prunus* spp. using aluminum cryo-plates. Sci. Hortic..

[98] Arizaga M.V., Yamamoto S.I., Tanaka D., Fukui K., Nohara N., Nishikawa T., Watanabe K.N. (2017). Cryopreservation of in vitro shoot tips of ulluco (*Ullucus tuberosus* Cal.) using D cryo-plate method. CryoLetters.

[99] Matsumoto T., Yamamoto S., Fukui K., Rafique T., Engelmann F., Niino T. (2015). Cryopreservation of persimmon shoot tips from dormant buds using the D cryo-plate technique. Hortic. J..

[100] Funnekotter B., Bunn E., Mancera R.L. (2017). Cryo-mesh: A simple alternative cryopreservation protocol. CryoLetters.

[101] Topal-Celikkan F., Ozkavukcu S., Balci D., Serin-Kilicoglu S., Atabenli-Erdemli E. (2015). Mouse ovarian tissue vitrification on copper electron microscope grids versus slow freezing: A comparative ultrastructural study. Reprod. Fertil. Dev..

[102] Reed B.M., Sarasan V., Kane M., Bunn E., Pence V.C. (2011). Biodiversity conservation and biotechnology tools. In Vitro Cell. Dev. Biol. Plant.

[103] Lambardi M., Benelli C., De Carlo A., Ozudogru E.A., Previati A., Ellis D. (2011). Cryopreservation of ancient apple cultivars of Veneto: A comparison between PVS2-vitrification and dormant-bud techniques. Acta Hortic..

[104] Fukui K., Shirata K., Niino T., Kashif I.M. (2011). Cryopreservation of mulberry winter buds in Japan. Int. Symp. Cryopreserv. Hortic. Species.

[105] Hirano T., Godo T., Mii M., Ishikawa K. (2005). Cryopreservation of immature seeds of *Bletilla striata* by vitrification. Plant Cell Rep..

[106] Jitsopakul N., Thammasiri K., Ishikawa K. (2008). Cryopreservation of *Bletilla striata* mature seeds, 3-day germinating seeds and protocorms by droplet-vitrification. CryoLetters.

[107] Antony J.J.J., Burkhan H., Sinniah U.R., Poobathy R., Subramaniam S. (2013). Effect of PVS2 vitrification on *Brassidium* shooting star orchid using protocorm-like bodies (PLBs). Aust. J. Crop Sci..

[108] Mubbarakh S.A., Rahmah S., Rahman Z.A., Sah N.N.M., Subramaniam S. (2014). Cryopreservation of *Brassidium* Shooting Star orchid using the PVS3 method supported with preliminary histological analysis. Appl. Biochem. Biotechnol..

[109] Negahdar N., Hashemabadi D., Kaviani B. (2021). In vitro conservation and cryopreservation of *Buxus sempervirens* L., a critically endangered ornamental shrub. Rus. J. Plant Physiol..

[110] Vettorazzi R.G., Silva Carvalho V., Carvalho Teixeira M., Campostrini E., Da Cunha M., de Monteiro Matos E., Facio Viccini L. (2019). Cryopreservation of immature and mature seeds of Brazilian orchids of the genus *Cattleya*. Sci. Hortic..

[111] Maneerattanarungroj P., Bunnag S., Monthatong M. (2007). In vitro conservation of *Cleisostoma areitinum* (Rchb.f.) Garay, rare Thai orchid species by an encapsulation-dehydration method. Asian J. Plant Sci..

[112] González-Benito M.E., Pérez C., Viviani A.B. (1997). Cryopreservation of nodal explants of an endangered plant species (*Centaurium rigualii* Esteve) using the encapsulation-dehydration method. Biodiver. Conserv..

[113] Gogoi K., Kumaria S., Tandon P. (2013). Cryopreservation of *Cymbidium eburneum* Lindl. and *C. hookerianum* Rchb.f., two threatened and vulnerable orchids via encapsulation–dehydration. In Vitro Cell. Dev. Biol. Plant.

[114] Surenciski M.R., Flachsland E.D., Terada G., Mroginski L.A., Rey H.Y. (2012). Cryopreservation of *Cyrtopodium hatschbachii* Pabst (Orchidaceae) immature seeds by encapsulation-dehydration. Biocell.

[115] Thammasiri K. (2008). Cryopreservation of some Thai orchid species. Acta Hort..

[116] Pimda W., Bunnag S. (2010). Cryopreservation of *Dendrobium heterocarpum* Lindl. via encapsulation-dehydration method. Elba Bioflux..

[117] Lurswijidjarus W., Thammasiri K. (2004). Cryopreservation of shoot tips of *Dendrobium* Walter Oumae by encapsulation/dehydration. Sci. Asia.

[118] Thammasiri K. (2000). Cryopreservation of seeds of a Thai orchid (*Doritis pulcherrima* Lindl.) by vitrification. CryoLetters.

[119] Seydi S., Sedaghathoor S., Kaviani B. (2020). In vitro storage techniques of *Fritillaria imperialis* Lubra Maxima, a wild rare and critically endangered ornamental species. CMU J. Nat. Sci..

[120] Mikuła A., Tomiczak K., Rybczýnski J.J. (2011). Cryopreservation enhances embryo-genic capacity of *Gentiana cruciata* (L.) suspension culture and maintains (epi)genetic uniformity of regenerants. Plant Cell Rep..

[121] Mikuła A., Fiuk A., Rybczýnski J.J. (2005). Induction, maintenance and preservation of embryogenic competence of *Gentiana cruciata* L. cultures. Acta Biol. Crac. Bot..

[122] Bouman H., Tiekstra A., Petutschnig E., Homan M., Schreurs R. (2003). Cryopreservation of *Lilium* species and cultivars. Acta Hortic..

[123] Yin Z.-F., Bi W.-L., Chen L., Zhao B., Wang Q.-C. (2014). An efficient, widely applicable cryopreservation of *Lilium* shoot tips by droplet-vitrification. Acta Physiol. Plant..

[124] Yi J.Y., Lee G.A., Chung J.W., Lee S.Y., Lim K.B. (2013). Efficient cryopreservation of *Lilium* spp. shoot tips using droplet-vitrification. Plant Breed. Biotech..

[125] Folgado R., Panis B. (2019). Cryopreservation of Ashe magnolia shoot-tips by droplet-vitrification. Acta Hortic..

[126] Lin L., Cai L., Fan L., Ma J.-C., Yang X.-Y., Hu X.-J. (2022). Seed dormancy, germination and storage behavior of *Magnolia sinica*, a plant species with extremely small populations of Magnoliaceae. Plant Divers..

[127] Kesa S., Limpanavech P., Kongchuensin S., Chareonsap P., Juthamas P. In vitro germplasm conservation of *Magnolia sirindhorniae* Noot. & Chalermglin in minimal growth condition and by cryopreservation. Proceedings of the 18th Annual Meeting of the Thai Society for Biotechnology. Biotechnology: Benefits & Bioethics.

[128] Miao N.H., Kaneko Y., Sugawara Y. (2005). Ultrastructural implications of pretreatment for successful cryopreservation of *Oncidium* protocorm-like body. CryoLetters.

[129] Flachsland E., Terada G., Scocchi A., Rey H., Mroginski L., Engelmann F. (2006). Cryopreservation of seeds and *in vitro*-cultured protocorms of *Oncidium bifolium* sims. (Orchidaceae) by encapsulation-dehydration. CryoLetters.

[130] Galdiano R.F.J., Lemos E.G.M., Faria R.T., Vendrame W.A. (2012). Cryopreservation of *Dendrobium* hybrid seeds and protocorms as affected by phloroglucinol and supercool X1000. Sci. Hortic..

[131] Hirano T., Godo T., Miyoshi K., Ishikawa K., Ishikawa M., Mii M. (2009). Cryopreservation and low-temperature storage of seeds of *Phaius tankervilleae*. Plant Biotechnol. Rep..

[132] Salaj T., Matusikova I., Fraterova L., Pirselova B., Salaj J. (2011). Regrowth of embryogenic tissues of *Pinus nigra* following cryopreservation. Plant Cell Tissue Org. Cult..

[133] Halmagyi A., Pinker I. (2006). Plant regeneration from *Rosa* shoot tips cryopreserved by a combined droplet vitrification method. Plant Cell Tissue Org. Cult..

[134] Pawłowska B., Bach A. (2011). Cryopreservation by encapsulation-dehydration of in vitro grown shoot buds of *Rosa* ‘New Dawn’. Acta Hortic..

[135] Jitsopakul N., Thammasiri K., Ishikawa K. (2009). Cryopreservation of *Vanda coerulea* protocorm-like bodies by droplet-vitrification. 1st International Symposium: Cryopreservation in Horticultural Species, Book of Abstract.

[136] Jitsopakul N., Thammasiri K., Ishikawa K. (2008). Cryopreservation of *Vanda coerulea* protocorms by encapsulation-dehydration. CryoLetters.

[137] Thammasiri K., Soamkul L. (2007). Cryopreservation of *Vanda coerulea* Griff. ex Lindl. seeds by vitrification. Sci. Asia.

[138] González-Arnao M.T., Lazaro-Vallejo C.E., Engelmann F., Gamez-Pastrana R., Martinez-Ocampo Y.M., Pastelin-Solano M.C., Diaz-Ramos C. (2009). Multiplication and cryopreservation of vanilla (*Vanilla planifolia* ‘Andrews’). In Vitro Cell. Dev. Biol.-Plant.

[139] Pathirana R., Mathew L., McLachlan A. (2021). A simplified method for high recovery of kiwifruit (*Actinidia* spp.) shoot tips after droplet vitrification cryopreservation suitable for long-term conservation. Plant Cell Tissue Organ Cult..

[140] Bachiri Y., Song G.Q., Plessis P., Shoar-Ghaffari A., Rekab T., Morisset C. (2001). Routine cryopreservation of kiwifruit (*Actinidia* spp) germplasm by encapsulation-dehydration: Importance of plant growth regulators. Cryoletters.

[141] Delgado-Aceves L., Portillo L., Folgado R., Romo-Paz F.J., González-Arnao M.T. (2022). New approaches for micropropagation and cryopreservation of *Agave peacockii*, an endangered species. Plant Cell Tissue Organ Cult..

[142] Delgado-Aceves L., González-Arnao M.T., Santacruz-Ruvalcaba F., Folgado R., Portillo L. (2021). Indirect somatic embryogenesis and cryopreservation of *Agave tequilana* Weber cultivar ‘Chato’. Plants.

[143] Souza F.V.D., Kaya E., Vieira L.J., Souza E.H., Amorim V.B.O., Skogerboe D., Matsumoto T., Alves A.A.C., da Silva Ledo C.A., Jenderek M.M. (2016). Droplet-vitrification and morphohistological studies of cryopreserved shoot tips of cultivated and wild pineapple genotypes. Plant Cell Tissue Org. Cult..

[144] da Silva R.L., de Souza E.H., Vieira L.d.J., Pelacani C.R., Souza F.V.D. (2017). Cryopreservation of pollen of wild pineapple accessions. Sci. Hortic..

[145] Souza F.V.D., Kaya E., de Jesus Vieira L., da Silva Souza A., de Jesus da Silva Carvalho M., Barbosa Santos E., Cunha Alves A.A., Ellis D. (2017). Cryopreservation of Hamilin sweet potato [(*Citrus sinensis* (L.) Osbeck)] embryonic calli using a modified aluminum cryo-plate technique. Sci. Hortic..

[146] Volk G.M., Bonnart R., Shepherd A., Yin Z.F., Lee L., Polek M., Krueger R. (2017). Citrus cryopreservation: Viability of diverse taxa and histological observations. Plant Cell Tissue Org. Cult..

[147] Wilms H., Rhee J.H., Rivera R.L., Longin K., Panis B. (2019). Developing coconut cryopreservation protocols and establishing cryogenebank at RDA; a collaborative project between RDA and biodiversity international. Acta Hortic..

[148] Niu Y.L., Luo Z.R., Zhang Y.F., Zhang Q.L. (2012). Cryopreservation of in vitro grown shoot tips of *Diospyros kaki* thunb. using different methods. CryoLetters.

[149] Matsumoto T., Niino T., Shirata K., Kurahashi T., Matsumoto S., Maki S. (2004). Long-term conservation of *Diospyros* germplasm using dormant buds by a prefreezing method. Plant Biotechnol..

[150] Ai P., Luo Z. (2005). Cryopreservation of dormant vegetative buds and genetic stability analysis of regenerated plantlets in persimmon. Acta Hortic..

[151] Benelli C., De Carlo A., Giordani E., Pecchioli S., Bellini E., Kochanova Z. (2009). Vitrification/one-step freezing procedure for cryopreservation of persimmon dormant buds. Acta Hortic..

[152] Ai P., Luo Z. (2003). Cryopreservation of dormant shoot-tips of persimmon by vitrification and plant regeneration. Sci. Agric. Sin..

[153] Matsumoto T., Mochida K., Itamura H., Sakai A. (2001). Cryopreservation of persimmon (*Diospyros kaki* Thunb.) by vitrification of dormant shoot tips. Plant Cell Rep..

[154] Panis B., Withers L.A., De Langhe E. (1990). Cryopreservation of *Musa* suspension cultures and subsequent regeneration of plants. CryoLetters.

[155] Generoso A.L., Carvalho V.S., Walter R., Campbell G., da Silva Araújo L., Silva Santana G., Cunha M. (2019). Mature-embryo culture in the cryopreservation of passion fruit (*Passiflora edulis* Sims) seeds. Sci. Hort..

[156] Garcia R.O., Pacheco G., Vianna M.G., Mansur E. (2011). In vitro conservation of *Passiflora suberosa* L., slow and cryopreservation. CryoLetters.

[157] Merhy T.S.M., Vianna M.G., Garcia R.O., Pacheco G., Mansur E. (2014). Cryopreservation of *Passiflora pohblii* nodal segments and assessment of genetic stability of regenerated plants. CryoLetters.

[158] O’Brien C., Hiti-Bandaralage J., Folgado R., Lahmeyer S., Hayward A., Folsom J., Mitter N. (2020). A method to increase regrowth of vitrified shoot tips of avocado (*Persea americana* Mill.): First critical step in developing a cryopreservation protocol. Sci. Hortic..

[159] O’Brien C., Parisi A., Lim Yiing Yuan J., Constantin M., Mitter N. (2016). Cryopreservation of avocado (*Persea americana* Mill.) using somatic embryos. Acta Hortic..

[160] Towill L.E., Forsline P.L. (1999). Cryopreservation of sour cherry (*Prunus ceraceus* L.) using a dormant vegetative bud method. CryoLetters.

[161] Shatnawi M.A., Shibli R., Qrunfleh I., Bataeineh K., Obeidat M. (2007). In vitro propagation and cryopreservation of *Prunus avium* using vitrification and encapsulation dehydration methods. J. Food Agric. Environ..

[162] Channuntapipat C., Collins G., Bertozzi T., Sedgley M. (2000). Cryopreservation of in vitro almond shoot tips by vitrification. J. Hort. Sci. Biotech..

[163] De Boucaud M.T., Brison M., Helliot B., Herve-Paulus V., Towill L.E., Bajaj Y.P.S. (2002). Cryopreservation in Prunus. Cryopreservation of Plant Germplasm II—Biotechnology in Agriculture and Forestry.

[164] Hao Y.J., Cheng Y.J., Deng X. (2005). Stable maintenance and expression of a foreign gene in transgenic pear shoots retrieved from in vitro conservation. J. Plant Physiol..

[165] Wang B., Yin Z.-F., Feng C.-H., Shi X., Li Y.-P., Wang Q.-C., Benkeblia N., Tennant P. (2008). Cryopreservation of potato shoot tips. Potato I Fruit, Vegetable and Cereal Science and Biotechnology, 2 (Special Issue 1).

[166] Chang Y., Reed B.M. (2001). Preculture conditions influence cold hardiness and regrowth of *Pyrus cordata* shoot tips after cryopreservation. HortScience.

[167] Chang Y., Reed B.M. (2000). Extended alternating-temperature cold acclimation and culture duration improve pear shoot cryopreservation. Cryobiology.

[168] Ren L., Wang M.R., Wang Q.C. (2021). ROS-induced oxidative stress in plant cryopreservation: Occurrence and alleviation. Planta.

[169] Ashburner M., Ball C.A., Blake J.A. (2000). Gene ontology consortium. Gene ontology: Tool for the unification of biology. Nat. Genet..

[170] Morrison N., Cochrane G., Faruque N., Tatusova T., Tateno Y., Hancock D., Field D. (2006). Concept of sample in omics technology. OMICS J. Integr. Biol..

[171] Harding K., Benson E.E., Normah N.M., Chin H.F., Reed B.M. (2012). Biomarkers from molecules to ecosystems and biobanks to genebanks. Conservation of Tropical Plant Species.

[172] Volk G.M. (2010). Application of functional genomics and proteomics to plant cryopreservation. Curr. Genom..

[173] Walters C., Wheeler L., Stanwood P.C. (2004). Longevity of cryogenically stored seeds. Cryobiology.

[174] Wang M.R., Bi W., Shukla M.R., Ren L., Hamborg Z., Blystad D.R., Saxena P.K., Wang Q.C. (2021). Epigenetic and genetic integrity, metabolic stability, and field performance of cryopreserved plants. Plants.

[175] Martinez-Montero M.E., González-Arnao M.T., Engelmann F., Katkov I.I. (2012). Cryopreservation of tropical plant germplasm with vegetative propagation—Review of sugarcane (*Saccharum* spp.) and pineapple (*Ananas comosus* (L.) Merrill) cases. Current Frontiers in Cryopreservation.

[176] Panis B., Van den Houwe I., Swennen R., Rhee J., Roux N. (2016). Securing plant genetic resources for perpetuity through cryopreservation. Indian J. Plant Genet. Resour..

[177] Seki M., Narusaka M., Ishida J. (2002). Monitoring the expression profiles of 7000 *Arabidopsis* genes under drought, cold, and high-salinity stresses using a full length cDNA microarray. Plant J..

[178] Takagi H., Engelmann F., Takai H. (2000). Recent development in cryopreservation of shoot apices of tropical species. Cryopreservation of Tropical Plant Germplasm Progress and Application.

[179] Fowler S., Thomashow M.F. (2002). Arabidopsis transcriptome profiling indicates that multiple regulatory pathways are activated during cold acclimation in addition to the CBF cold response pathway. Plant Cell.

[180] Kim C.Y., Liu Y., Thorne E.T., Yang H., Fukushige H., Gassmann W., Hildebrand D., Sharp R.E., Zhang S. (2003). Activation of a stress-responsive mitogen activated protein kinase cascade induces the biosynthesis of ethylene in plants. Plant Cell.

[181] Lynch P.T., Siddika A., Johnston J.W., Mehra A., Benelli C., Lambardi M., Benson E.E. (2011). Effects of osmotic pretreatments on oxidative stress and antioxidant profiles of cryopreserved olive somatic embryos. Plant Sci..

[182] Grapin A., Dorion N., Verdeil J.L., Escoute J. (2007). Histo-cytological changes in *Pelargonium* apices during the cryopreservation process: Effect of the osmotic agent chosen for the preculture step. Acta Hortic..

[183] Gamez-Pastrana R., Gonzalez-Arnao M.T., Martinez-Ocampo Y., Engelmann F. (2011). Thermal events in calcium alginate beads during encapsulation dehydration and encapsulation-vitrification protocols. Acta Hortic..

[184] Yang Z., Sheng J., Lv K., Ren L., Zhang D. (2019). Y2SK2 and SK3 type dehydrins from *Agapanthus praecox* can improve plant stress tolerance and act as multifunctional protectants. Plant Sci..

[185] Chen G.Q., Ren L., Zhang D., Shen X.H. (2016). Glutathione improves survival of cryopreserved embryogenic calli of *Agapanthus praecox* subsp. orientalis. Acta Physiol. Plant..

[186] Di W., Jiang X., Xu J., Jia M., Li B., Liu Y. (2018). Stress and damage mechanisms in *Dendrobium nobile* Lindl. protocorm-like bodies during pre- and post-liquid nitrogen exposure in cryopreservation revealed by iTRAQ proteomic analysis. In Vitro Cell. Dev. Biol. Plant.

[187] Jiang X., Ren R., Di W., Jia M., Li Z., Liu Y., Gao R. (2019). Hydrogen peroxide and nitric oxide are involved in programmed cell death induced by cryopreservation in *Dendrobium* protocorm-like bodies. Plant Cell Tissue Organ Cult..

[188] Poobathy R., Sinniah U.R., Xavier R., Subramaniam S. (2013). Catalase and superoxide dismutase activities and the total protein content of protocorm-like bodies of Dendrobium Sonia-28 subjected to vitrification. Appl. Biochem. Biotechnol..

[189] Rahmah S., Mubbarakh A., Ping S., Subramaniam K.S. (2015). Effects of droplet-vitrification cryopreservation based on physiological and antioxidant enzyme activities of Brassidium shooting star orchid. Sci. World J..

[190] Antony J.J.J., Zakaria S., Zakaria R., Ujang J.A., Othman N., Subramaniam S. (2019). Biochemical analyses of *Dendrobium* Sabin Blue PLBs during cryopreservation by vitrification. Physiol. Mol. Biol. Plant.

[191] Vianna M.G., Garcia R.O., Mansur E., Engelmann F., Pacheco G. (2019). Oxidative stress during the cryopreservation of *Passiflora suberosa* L. shoot tips using the V-Cryo-plate technique: Determination of the critical stages of the protocol. Plant Cell Tissue Organ Cult..

[192] Johnston J.W., Benson E.E., Harding K. (2009). Cryopreservation of in vitro *Ribes* shoots induces temporal changes in DNA methylation. Plant Physiol. Biochem..

[193] Castillo N.R.F., Bassil N.V., Wada S., Reed B.M. (2010). Genetic stability of cryopreserved shoot tips of *Rubus* germplasm. In Vitro Cell. Dev. Biol. Plant.

[194] Maki S., Hirai Y., Niino T., Matsumoto T. (2015). Assessment of molecular genetic stability between long-term cryopreserved and tissue cultured wasabi (*Wasabia japonica*) plants. CryoLetters.

[195] Li J.-W., Ozudogru E.A., Li J., Wang M.-R., Bi W.-L., Lambardi M., Wang Q.-C. (2017). Cryobiotechnology of forest trees: Recent advances and future prospects. Biodivers. Conserv..

[196] IUCN (2016). The IUCN Red List of Threatened Species.

